# *NR5A1* gene variants in infertile Senegalese men: Discovery of a novel missense variant and genotype-phenotype correlation^☆^

**DOI:** 10.1016/j.jgeb.2025.100578

**Published:** 2025-09-27

**Authors:** Adji Dieynaba Diallo, Arame Ndiaye, Ndiaga Diop, Fatou Diop Gueye, Mame Venus Gueye, Yacouba Dia, Amath Thiam, Abdoulaye Séga Diallo, Rokhaya Ndiaye, Oumar Faye, Mama Sy

**Affiliations:** a Doctoral School of Life, Health and Environmental Sciences, ED-SEV, Biology and Human Pathologies, Faculty of Medicine, Pharmacy and Odontology, Cheikh Anta Diop University, Dakar, Senegal; b Clinical Cytology, Cytogenetics, Reproductive Biology and Human Development Laboratory, Aristide Le Dantec Hospital, Dakar, Senegal; c Immunogenetic Laboratory Faculty of Medicine, Pharmacy and Odontology, Cheikh Anta Diop University, Dakar, Senegal; d Urology Department, Ouakam Military Hospital, Dakar, Senegal

**Keywords:** *NR5A1*, Infertility, Senegal, SF-1, Variants, DSD, Genomics

## Abstract

The *NR5A1* gene, encoding Steroidogenic Factor 1 (SF-1), plays a critical role in sex differentiation and spermatogenesis. However, data on *NR5A1* variants in sub-Saharan African populations remain limited. This exploratory pilot study aimed to identify and characterize *NR5A1* variants in infertile Senegalese men and to assess genotype–phenotype correlations. We conducted a cross-sectional study in 23 infertile Senegalese men, and exons 2 –7 of *NR5A1* were sequenced using the Sanger method. Detected variants were analyzed with in silico prediction tools and filtered for rarity (minor allele frequency < 1 %) using the gnomAD database. Genotype–phenotype associations were analyzed using Fisher’s exact test.

Eighty-three percent of patients harbored at least one *NR5A1* variant. A novel heterozygous missense variant, c.584C > T (p.Ser195Phe), located in the hinge region of SF-1, was identified in three unrelated individuals presenting with micropenis, testicular hypotrophy, and azoospermia. Several previously described variants were also detected. Genotype–phenotype correlation analysis revealed significant associations between *NR5A1* variants and spermatogenic failure, the most common clinical feature (60.8 %). These findings demonstrate substantial genetic variability in *NR5A1* among Senegalese men with infertility, and identify a novel missense variant. Our study highlights the relevance of *NR5A1* in male infertility screening and emphasizes the importance of incorporating African populations into reproductive genetics research.

## Highlights

• Identified 26 *NR5A1* variants in 23 infertile Senegalese men.
• Discovered novel missense variant c.584C > T (p.Ser195Phe) linked to infertility.
• 83% of patients carried at least one *NR5A1* variant, highlighting high prevalence.
• Significant association found between *NR5A1* variants and spermatogenic failure.
• UpSet plot revealed phenotype clusters: micropenis, testicular hypotrophy, azoospermia.

## Graphical abstract

**Figure:**
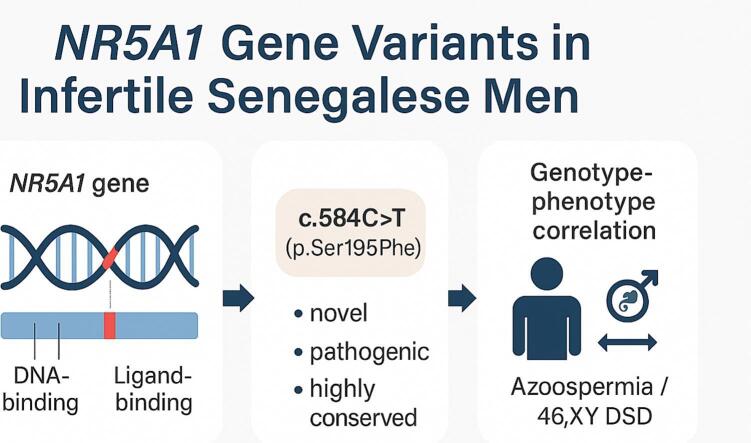

## 1 Introduction

Infertility affects approximately 17.5 % of couples of reproductive age worldwide^1^. In sub-Saharan Africa, prevalence ranges from 11 % to 25 %, with studies in Senegal indicating that 15–20 % of couples seek medical care for infertility.^2^ Despite this burden, infertility is often perceived primarily as a female issue, although male factors contribute to nearly 50 % of cases. Male infertility is frequently associated with hormonal imbalances, impaired spermatogenesis, and reduced reproductive capacity.^3^, ^4^

The etiology of male infertility is heterogeneous and multifactorial, including anatomical abnormalities, environmental exposures, infections, and increasingly recognized genetic factors. Genetic causes are estimated to account for 10–15 % of male infertility cases,^5^ with chromosomal anomalies and Y chromosome microdeletions being the most frequently identified. Monogenic defects in genes involved in sex determination and steroidogenesis are gaining attention.

Among these, *NR5A1* (nuclear receptor subfamily 5 group A member 1), located on chromosome 9q33.3, encodes Steroidogenic Factor 1 (SF-1), a 461-amino-acid transcription factor essential for adrenal and gonadal development and for the regulation of steroid biosynthesis.^6^ In the testes, SF-1 activates transcription of *SOX9* gene, crucial for Sertoli cell differentiation,^7^, ^8^ and promotes anti-Müllerian hormone (*AMH*) expression, facilitating Müllerian ducts regression during male sexual differentiation.^9^, ^10^ SF-1 also regulates testosterone synthesis in Leydig cells, contributing to the development of male internal and external genitalia.^11^, ^12^ In females, SF-1 is expressed in granulosa and theca cells, regulating steroid hormone production.^13^

The first human *NR5A1* mutation was reported in a 46,XY individual with adrenal insufficiency and complete gonadal dysgenesis.^14^ Since then, the phenotypic spectrum has expanded to include 46,XY and 46,XX disorders of sex development (DSD), isolated male infertility, primary ovarian insufficiency (POI), and even asymptomatic carriers.^15^, ^16^ According to the Human Gene Mutation Database (HGMD), over 218 pathogenic or likely pathogenic *NR5A1* variants have been identified.^17^ These include missense, nonsense, frameshift, splice-site, insertion, deletion, and complex rearrangements across the coding region. Although most variants are heterozygous, the wide phenotypic variability suggests the involvement of additional genetic, epigenetic, or environmental modifiers.^18^

Recent studies have highlighted the pleiotropic nature of *NR5A1* mutations. Elzenaty et al.^19^ reported 35 novel *NR5A1* variants in individuals with DSD, highlighting the gene’s critical role in gonadal development and adrenal function. Similarly, Kouri et al.^20^ investigated a large international cohort and emphasized the contribution of *NR5A1* variants to both 46,XY DSD and isolated male infertility.

Despite evidence from Europe, Asia, and the Americas, data from African populations remain scarce, particularly in West Africa. This lack of representation limits the development of inclusive genetic screening tools and hampers equitable diagnostic and therapeutic approaches on a global scale. In this context, studying *NR5A1* variants in Senegalese men offers a unique opportunity to broaden current understanding of genotype–phenotype relationships by incorporating data from an underrepresented population with distinct genetic backgrounds.

Given the paucity of data on *NR5A1* variants in the Senegalese population, we conducted this exploratory pilot study to assess the feasibility of targeted genetic screening in this context. Our primary objective was to identify rare *NR5A1* variants and generate preliminary evidence to guide future research on the genetic basis of male infertility in West Africa.

## 2 Materials and methods

### 2.1 Study population

This cross-sectional pilot study included 23 Senegalese men aged 21–45 years, all presenting with primary infertility. Participants were referred to the Laboratory of Clinical Cytology, Cytogenetics, Reproductive Biology, and Human Development at Aristide Le Dantec Hospital, Dakar, for evaluation.

#### 2.1.1 Inclusion and exclusion criteria

Inclusion and exclusion criteria were prospectively defined prior to enrollment.

**Inclusion criteria** were as follows:

– Men aged 21– 45 years.
– Primary infertility, defined as the failure to achieve a clinical pregnancy after ≥ 12 months of regular unprotected intercourse, according to WHO 2021 guidelines (6th edition).^1^
– Abnormal semen parameters were defined based on WHO 2010 reference values (5th edition),^21^ which were the standard at the time of data collection.
– 46,XY karyotype.

**Exclusion criteria** were as follows:

– Prior chemotherapy or radiotherapy.
– Known chromosomal abnormalities, except for suspected or confirmed disorders of sex development (DSD).
– Obstructive azoospermia.
– History of genitourinary surgery affecting fertility.
– Active genitourinary infection at the time of sampling.

#### 2.1.2 DSD features and controls

**DSD features**

A subset of participants presented clinical features suggestive of disorders of sex development, such as hypospadias, micropenis, or undescended testes.

**Controls**

Two fertile Senegalese men with confirmed 46,XY karyotypes, normozoospermia, and at least one naturally conceived child were included in the study and considered as negative controls. Given the small sample size, they served as a qualitative reference for comparative genetic analysis.

### 2.2 E thical approval

This study was approved by the Research Ethics Committee of Cheikh Anta Diop University (CER/UCAD/AD/MsN/014/2020, dated February 17, 2021) in Dakar, Senegal. The research was conducted in accordance with the Declaration of Helsinki and adhered to national and institutional ethical guidelines. Written informed consent was obtained from all participants after providing a clear explanation of the study's objectives, procedures, and potential risks.

### 2.3 Clinical, laboratory, and molecular assessment

All participants underwent a standardized clinical, laboratory, and molecular evaluation, including medical and reproductive history, physical examination, semen analysis, hormonal profiling, and *NR5A1* gene sequencing.

#### 2.3.1 Clinical and laboratory assessment

Clinical assessment included a detailed medical and reproductive history, as well as a physical examination with measurement of testicular volume using a Prader orchidometer and evaluation for genital anomalies, including hypospadias, undescended testes, or micropenis. Laboratory assessment comprised semen analysis of two separate samples, performed according to the WHO 2010 guidelines, which were the reference standard at the time of data collection. Serum follicle-stimulating hormone (FSH), luteinizing hormone (LH), and total testosterone levels were also measured.

#### 2.3.2 Molecular assessment

Genomic DNA was extracted from peripheral blood samples using the Quick-DNA MiniPrep Kit (Zymo Research), in accordance with the manufacturer's instructions. The coding regions of the *NR5A1* gene (exons 2–7; reference sequence NM_004959.5) were individually amplified by polymerase chain reaction (PCR). Each 25 μL PCR reaction contained: 12.5 μL of 2 × OneTaq® Quick-Load® Master Mix, 0.5 μL of each primer (10 μM), 2 μL of genomic DNA (≈50 ng/μL), and 7.5 μL of Milli-Q water. Thermal cycling conditions were as follows: initial denaturation at 94 °C for 2 min; 35 cycles of 94 °C for 1 min, exon-specific annealing at 57–60 °C for 1 min, and 72 °C for 1 min; followed by a final extension at 72 °C for 10 min. Primer sequences, amplicon sizes, and exon-specific PCR conditions are provided in Supplementary Table S1. Primers were adapted from Sudhakar et al.^22^ and synthesized by Incaba Biotec (South Africa). PCR products from all patients and controls were purified and sequenced by Sanger across the entire coding region of *NR5A1* at the Eurofins Genomics platform.

### 2.4 Bioinformatic analysis

The raw sequencing files (FASTQ format) were reviewed, quality-checked, and aligned using BioEdit software (v7.2.5). Variant calling was performed with Mutation Surveyor® (v5.1.2) and DNA Baser (v5.0), using the GRCh37 human genome reference assembly. All variants were annotated following the Human Genome Variation Society (HGVS) nomenclature and classified according to the 2015 ACMG/AMP guidelines issued by the American College of Medical Genetics.

Identified variants were cross-referenced with public databases, including dbSNP, the Human Gene Mutation Database (HGMD), and Ensembl Genome Browser (v110). Minor allele frequencies (MAF) were extracted from the Genome Aggregation Database (gnomAD). Functional predictions were generated using a panel of in silico tools, including SIFT, PolyPhen-2, REVEL, M-CAP, PANTHER, E-SNPs&GO, Mutation Assessor, SNPs&GO, and MutationTaster. Only variants with a MAF below 1 % in gnomAD were considered for downstream analysis.

All sequence variants identified in this study have been deposited in GenBank under the following accession numbers: OR834987, OR892276 to OR892296.

### 2.5 Variant filtering

A stringent filtering strategy was applied to all detected variants. Variants were retained if they met the following criteria: (1) localization within coding exons, (2) minor allele frequency (MAF) < 1 % in the gnomAD database, and (3) classification as pathogenic or potentially deleterious based on in silico prediction tools.

### 2.6 Statistical analysis

Associations between the presence of *NR5A1* variants and clinical phenotypes (including spermatogenic failure, azoospermia, severe oligozoospermia, cryptozoospermia, and DSD features) were assessed within the patient cohort. Given the limited sample size, Fisher’s exact test was used for all categorical analyses. Control subjects were not included in inferential statistics; Fisher’s exact tests were restricted to patient subgroups. Effect sizes are reported as odds ratios (OR) with 95 % confidence intervals (CI) when estimable. Odds ratios and exact 95 % CIs were computed using the fisher.test function in R, and all tests were two-sided. A p-value < 0.05 was considered statistically significant. No corrections for multiple testing were applied due to the exploratory nature of this pilot study. The primary outcome of interest was the association between *NR5A1* variants and spermatogenic failure, while secondary analyses explored associations with specific semen phenotypes and DSD features. All statistical analyses were performed using R software (v4.5.0). Heatmaps were generated with the ComplexHeatmap package, and UpSet plots were created using the UpSetR package.

### 2.7 AI usage statement

Generative AI tools (ChatGPT, DeepL translate) were used exclusively for language editing. No part of the study design, data analysis, or scientific interpretation was generated using AI.

## 3 Results

### 3.1 Clinical characteristics

The 23 infertile men included in this pilot study (Table 1) had a mean age of 31.1 ± 6.5 years. The majority presented with spermatogenic failure (azoospermia or severe oligozoospermia; n = 19), while 4 patients had cryptozoospermia.

**Table 1:** Clinical and Demographic Characteristics of Senegalese Men with Infertility.

ID	Age (years)	Ethnicity	Clinical Signs	Semen category	Hormonal Profile			Karyotype	Phenotype	Severity grade
					FSH (IU/L)	LH (IU/L)	Total T (ng/mL)			
1	32	Halpulaar	Testicular hypotrophy	Azoospermia	33.80	17.71	3.24	46,XY	Spermatogenic failure with testicular hypotrophy	Severe
2	29	Ouolof	Gynecomastia; Testicular hypotrophy	Azoospermia	64	68	19.8	46,XY	Spermatogenic failure with androgen axis alteration	Very severe
3	40	Ouolof	Testicular hypotrophy	Azoospermia	14.71	NR	12.75	46,XY	Spermatogenic failure; family history positive	Severe
4	34	Halpulaar	Gynecomastia; Hypospadias; Testicular hypotrophy; Micropenis	Azoospermia	58.28	NR	14.78	47,XXY/46,XX	DSD features with spermatogenic failure (mosaic karyotype)	Very severe
5	30	Ouolof	Normal genital exam	Azoospermia	NR	NR	NR	46,XY	Spermatogenic failure	Severe
6	30	Halpulaar	Normal genital exam	Cryptozoospermia	NR	NR	NR	46,XY	Spermatogenic failure (mild)	Moderate
7	30	Diola	Testicular hypotrophy	Azoospermia	27.60	19.15	15.26	46,XY	Spermatogenic failure with testicular hypotrophy	Severe
8	30	Ouolof	Testicular hypotrophy	Azoospermia	23.68	NR	5.73	46,XY	Spermatogenic failure with testicular hypotrophy	Severe
9	44	Sérère	Normal genital exam	Severe oligozoospermia	39.70	NR	6.82	46,XY	Spermatogenic failure	Severe
10	32	Sérère	Normal genital exam	Cryptozoospermia	NR	NR	NR	46,XY	Spermatogenic failure (mild)	Moderate
11	22	Sarakhole	Normal genital exam	Azoospermia	NR	NR	NR	46,XY	Spermatogenic failure	Severe
12	31	Halpulaar	Testicular hypotrophy	Cryptozoospermia	32.8	NR	8.8	46,XY	Spermatogenic failure (mild)	Moderate
13	39	Halpulaar	Normal genital exam	Azoospermia	NR	NR	NR	46,XY	Spermatogenic failure	Severe
14	45	Diola	Normal genital exam	Severe oligozoospermia	NR	NR	NR	46,XY	Spermatogenic failure	Severe
15	23	Bambara	Normal genital exam	Azoospermia	NR	NR	NR	46,XY	Spermatogenic failure	Severe
16	23	Halpulaar	Normal genital exam	Severe oligozoospermia	16.2	NR	8.8	46,XY	Spermatogenic failure	Severe
17	25	Ouolof	Normal genital exam	Azoospermia	41.73	11.4	1.34	46,XY	Spermatogenic failure with hypergonadotropic profile	Very severe
18	29	Diola	Normal genital exam	Cryptozoospermia	NR	NR	NR	46,XY	Spermatogenic failure (mild)	Moderate
19	30	Sérère	Normal genital exam	Azoospermia	39.70	NR	1.42	46,XY	Spermatogenic failure with hypergonadotropic profile	Very severe
20	28	Sérère	Normal genital exam	Azoospermia	29.56	17.44	2.04	46,XY	Spermatogenic failure with elevated FSH/LH	Very severe
21	21	Ouolof	Testicular hypotrophy; Gynecomastia	Azoospermia	24.34	18.42	0.35	47,XXY/46,XY	DSD features with spermatogenic failure	Very severe
22	38	Sérère	Testicular hypotrophy; Micropenis	Azoospermia	10.71	NR	16.51	47,XXY/46,XY	DSD features with spermatogenic failure	Very severe
23	30	Ouolof	Testicular hypotrophy; Inguinal hernia	Azoospermia	23.62	24.45	0.68	47,XXY/46,XY	DSD features with spermatogenic failure	Very severe

NR: Not reported.

On clinical examination, testicular hypotrophy was observed in 10 patients, gynecomastia in 3, micropenis in 2, hypospadias in 1, and inguinal hernia in 1. Four patients had karyotypes consistent with disorders of sex development (DSD; 47,XXY/46,XY mosaicism or 47,XXY/46,XX), all of whom exhibited very severe spermatogenic failure.

Hormonal profiles were variable, with markedly elevated FSH and LH in patients with DSD and hypergonadotropic features. Severity grading indicated that 11 patients were classified as “Severe”, 8 “Very severe,” while 4 showed moderate impairment.

The two fertile controls (mean age 30.0 ± 2.8 years) had normal genital examinations, normozoospermia, and hormone levels within the reference range (Supplementary Table S2).

### 3.2 Identification and distribution of NR5A1 variants

**Fig. 1:**
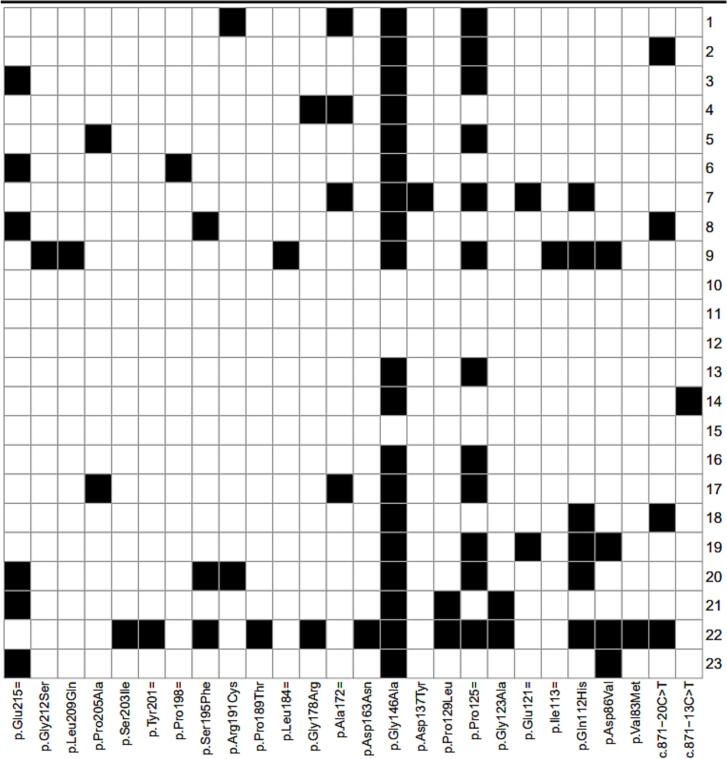

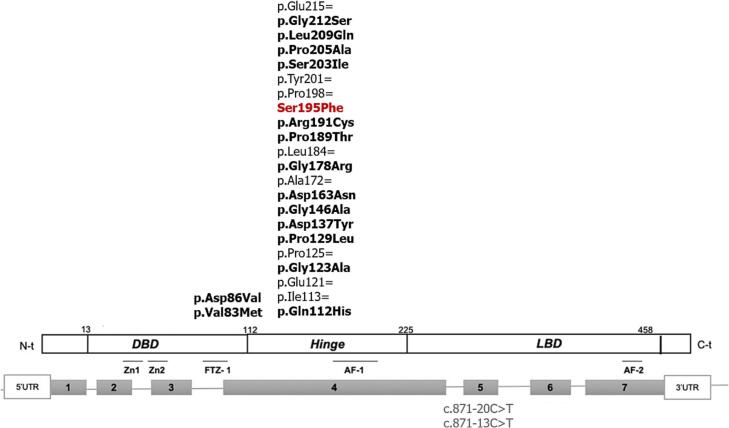
Overview and visualization of *NR5A1* variants identified in the present study. **(a)** Heatmap illustrating the frequency and distribution of each *NR5A1* variants among the 23 patients included in the study. Each row represents a patient, and each column corresponds to a specific variant position. **(b)** Positional mapping of *NR5A1* variants across functional domains of the SF-1 protein. Variants were mapped to three main domains: the DNA-binding domain (DBD), the hinge region, and the ligand-binding domain (LBD).

A total of 26 distinct *NR5A1* gene variants were identified among the 23 infertile Senegalese men analyzed (Fig. 1a). All identified variants were also screened in the two control individuals, and none of the predicted pathogenic variants were present.

Following variant analysis, 92.3 % (24/26) of these variants were located in exon 4, which encodes the DNA-binding domain (DBD) and the hinge region of the SF-1 protein (Fig. 1b). These variants included both synonymous and non-synonymous substitutions and were predominantly found in the heterozygous state.

Supplementary Table S3 provides a comprehensive overview of all identified variants, including HGVS nomenclature, predicted functional effects, ACMG classification, ClinVar annotation, and multiple in silico predictions.

Genomic positions and allele frequencies in the cohort and in gnomAD populations are provided in Supplementary Table S4.

### 3.3 Patients without *NR5A1* variants

Among the 23 infertile men, four patients (IDs 10, 11, 12 and15) did not carry any *NR5A1* variant. Clinically, these patients presented with azoospermia (n = 3) or cryptozoospermia (n = 1) and exhibited normal genital examination without major anomalies. Two of the four patients had reduced testicular volume. Hormonal profiles showed FSH and LH values within the upper normal range or mildly elevated (FSH: 16.2–32.8 IU/L; LH: 8.8–17.4 IU/L), with total testosterone levels mostly within reference intervals. Karyotypes were all 46,XY. Compared to patients harboring *NR5A1* pathogenic or likely pathogenic variants, these individuals showed similar spermatogenic impairment but tended to have slightly lower gonadotropin elevations. These observations suggest that, in addition to *NR5A1* variants, other genetic or environmental factors may contribute to spermatogenic failure in this cohort (see Supplementary Table S5 for detailed characteristics).

3.4 Discovery of a novel Missense variant and co-occurring *NR5A1* variants

A novel heterozygous missense variant, c.584C > T (p.Ser195Phe), was identified in three unrelated patients. All three individuals presented overlapping clinical features, including micropenis, testicular hypotrophy, and spermatogenic failure. The variant is located within a highly conserved region of the SF-1 hinge domain and was absent from major public databases (gnomAD, dbSNP, HGMD). Multiple in silico prediction tools classified this variant as potentially deleterious.

**Fig. 2:**
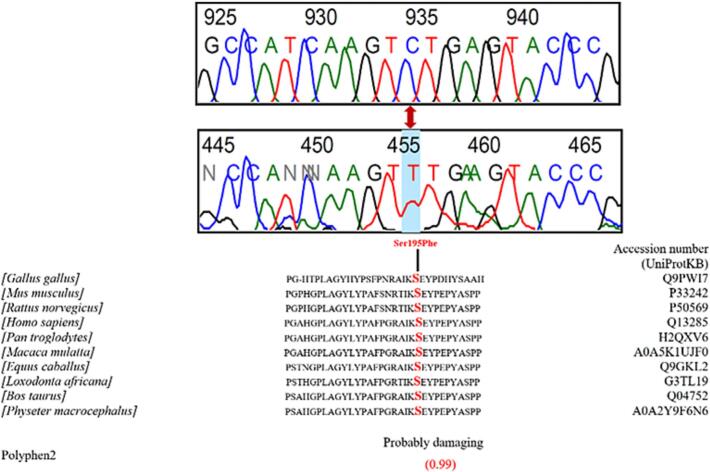
Molecular analysis of the novel *NR5A1* variant c.584C > T (p.Ser195Phe). Sanger sequencing chromatogram showing the heterozygous c.584C > T substitution (red arrow). A multiple sequence alignment highlights strong evolutionary conservation of the affected amino acid residue (Ser195, shown in red) across several vertebrate species, supporting its potential functional relevance. (For interpretation of the references to colour in this figure legend, the reader is referred to the web version of this article.)

Molecular analysis further supported the pathogenic potential of this variant. The Sanger sequencing chromatogram confirmed the heterozygous substitution (Fig. 2), and multiple sequence alignment demonstrated the evolutionary conservation of the Ser195 residue across species (Fig. 2).

Among the three patients, one individual carried the novel c.584C > T variant alone, while the other two also harbored additional *NR5A1* variants. Table 2 summarizes the novel variant and its co-occurrence with other pathogenic or likely pathogenic variants. These co-occurring variants have been previously reported as pathogenic or likely pathogenic, suggesting that their presence may contribute cumulatively or modify the severity of the spermatogenic phenotype. The overall distribution of *NR5A1* variants in the cohort, including single and co-occurring variants, is illustrated in Fig. 1a (heatmap), highlighting their potential role in phenotypic variability.

**Table 2:** Pathogenic and Novel *NR5A1* Variants Identified in Patients Carrying the p.Ser195Phe variant.

Patient ID	HGVSc	HGVSp	ACMG Class.	Clinvar	In silico Predictions								Additional Variants Present	Associated Phenotype
					SIFT	PolyPhen-2	MutationTaster	REVEL	E-SNPs&GO	M−CAP	Panther	SNPs&Go		
8	0c.584C > T	p.Ser195Phe	VUS	Novel	Del	Prdam	P	ND	P	P	Prdam	Neu	None	Spermatogenic failure with testicular hypotrophy
20	0c.584C > T	p.Ser195Phe	VUS	Novel	Del	Prdam	P	ND	P	P	Prdam	Neu	0c.571C > T, p.Arg191Cys; c.336G > T, p.Gln112His	Spermatogenic failure with elevated FSH/LH
22	0c.584C > T	p.Ser195Phe	VUS	Novel	Del	Prdam	P	ND	P	P	Prdam	Neu	0c.565C > T, p.Pro189Thr; 0c.532G > A, p.Gly178Arg; 0c.386C > T, p.Pro129Leu; 0c.368G > C, p.Gly123Ala; 0c.336G > T, p.Gln112His; 0c.257A > T, p.Asp86Val; 0c.247G > A, p.Val83Met	DSD features with spermatogenic failure, Testicular hypotrophy; Micropenis

ACMG, American College of Medical Genetics and Genomics Classification; B, Benign; Dis, disease; Het, Heterozygous; Hom, Homozygous; LB, Likely Benign; LP, Likely Pathogenic; ND, Not Defined; Neu, Neutral; P, Pathogenic; Del, Deleterious; T, Tolerated; Prdam, probably damaging; VUS, Variant of unknown significance; In silico analysis with webtool: SIFT (https://sift.bii.a-star.edu.sg/), PolyPhen-2 (https://genetics.bwh.harvard.edu/pph2/), MutationTaster (https://www.genecascade.org/MutationTaster2021/#transcript), Panther (https://pantherdb.org/), SNPs&Go (https://snps-and-go.biocomp.unibo.it/snps-and-go/), M-CAP (https://bejerano.stanford.edu/mcap/), REVEL (https://sites.google.com/site/revelgenomics/), E-SNPs&GO (https://esnpsandgo.biocomp.unibo.it/) *NR5A1*: NM_004959.5.

### 3.4 Filtering and classification of potentially deleterious variants

After applying the established filtering criteria, eleven *NR5A1* variants were retained for further analysis based on their predicted pathogenicity. These retained variants, along with their predicted pathogenicity and in silico annotations (SIFT, PolyPhen-2, MutationTaster, etc.), are summarized in Table 3. This set comprises the novel c.584C > T (p.Ser195Phe) variant identified in this cohort, seven previously reported variants with documented clinical relevance, and three additional variants (c.409G > T, c.336G > T, c.257A > T) present in public databases but not yet described in peer-reviewed studies. The potential pathogenicity of these database-only variants is supported by multiple in silico prediction tools. The clinical associations of these variants within the study cohort are detailed in Table 4.

**Table 3:** Molecular characteristics and in silico predictions of *NR5A1* variants retained after ACMG classification.

HGVSc	HGVSp	Variant effect	Rs number	ACMG Class.	Clinvar	SIFT	PolyPhen-2	MutationTaste	REVEL	E-SNPs&GO	M-CAP	Panther	SNPs&Go
0c.634G > A	p.Gly212Ser	Missense	rs201095702	**P**	**P**	T	B	P	likely disease causing	B	P	Posdam	Neu
0c.584C > T	p.Ser195Phe	Missense	Novel variant	**VUS**	ND	Del	Prdam	P	ND	P	P	Prdam	Neu
0c.571C > T	p.Arg191Cys	Missense	rs1253324106	**P**	ND	ND	Posdam	P	likely disease causing	P	P	Prdam	Dis
0c.565C > T	p.Pro189Thr	Missense	rs1483691434	**LP**	**LP**	ND	B	B	LB	B	P	Posdam	Neu
0c.409G > T	p.Asp137Tyr	Missense	rs745949372	**VUS**	ND	ND	Posdam	B	LB	P	P	Prdam	Neu
0c.532G > A	p.Gly178Arg	Missense	rs543895681	**LP**	ND	ND	Prdam	B	LB	B	P	Posdam	Dis
0c.386C > T	p.Pro129Leu	Missense	rs200749741	**P**	**P**	T	B	B	LB	B	P	Posdam	Neu
0c.368G > C	p.Gly123Ala	Missense	rs200163795	**P**	**P**	T	B	B	LB	B	P	Posdam	Neu
0c.336G > T	p.Gln112His	Missense	rs1564152710	**VUS**	ND	ND	B	B	LB	P	P	Prben	Neu
0c.257A > T	p.Asp86Val	Missense	rs751670386	**VUS**	ND	ND	Prdam	P	LB	P	P	Prdam	Dis
0c.247G > A	p.Val83Met	Missense	rs1832458349	**VUS**	ND	ND	Prdam	P	ND	P	P	Prdam	Dis

ACMG, American College of Medical Genetics and Genomics Classification; LP, Likely Pathogenic; P, Pathogenic; VUS, Variant of unknown significance; ND, Not Defined; T, Tolerated; Del, Deleterious; Prdam, Probably damaging; Posdam, Possibly damaging; B, Benign; LB, Likely benign; Prben, Probably benign; Neu, Neutral; Dis, Disease causing; In silico analysis with webtool: SIFT (https://sift.bii.a-star.edu.sg/), PolyPhen-2 (https://genetics.bwh.harvard.edu/pph2/), MutationTaster (https://www.genecascade.org/MutationTaster2021/#transcript), Panther (https://pantherdb.org/), SNPs&Go (https://snps-and-go.biocomp.unibo.it/snps-and-go/), M-CAP (https://bejerano.stanford.edu/mcap/), REVEL (https://sites.google.com/site/revelgenomics/), E-SNPs&GO (https://esnpsandgo.biocomp.unibo.it/) *NR5A1*: NM_004959.5.

**Table 4:** Clinical phenotypes associated with *NR5A1* variants in a senegalese infertile cohort compared with previously reported phenotypes.

dbSNP RSID	HGVSc	HGVSp	Patient phenotype	Reported phenotype	Reference
rs201095702	0c.634G > A	p.Gly212Ser	Spermatogenic failure	Spermatogenic failure 8	23, 24
	**0c.584C > T**	**p.Ser195Phe**	Spermatogenic failure; 47,XXY/46,XY; Testicular hypotrophy; Micropenis	Not previously reported (novel)	
rs1253324106	0c.571C > T	p.Arg191Cys	Spermatogenic failure; Testicular hypotrophy	46,XY sex reversal 3	^23^
rs1483691434	0c.565C > A	p.Pro189Thr	Spermatogenic failure; 47,XXY/46,XY; Testicular hypotrophy; Micropenis	Genetic non-acquired premature ovarian failure	^25^
rs543895681	0c.532G > A	p.Gly178Arg	Spermatogenic failure; 46,XX/47,XXY; Gynecomastia; Testicular hypotrophy; Hypospadias; Micropenis;	XY gonadal dysgenesis	^26^
rs745949372	0c.409G > T	p.Asp137Tyr	Spermatogenic failure; Testicular hypotrophy	Not reported in ClinVar	
rs200749741	0c.386C > T	p.Pro129Leu	Spermatogenic failure; 47,XXY/46,XY; Testicular hypotrophy; Gynecomastia; Micropenis	Premature ovarian failure 7; Spermatogenic failure 8	23, 27, 28
rs200163795	0c.368G > C	p.Gly123Ala	Spermatogenic failure; 47,XXY/46,XY; Testicular hypotrophy; Gynecomastia; Micropenis	Premature ovarian failure 7; Spermatogenic failure 8	23, 27, 28
rs1564152710	0c.336G > T	p.Gln112His	Spermatogenic failure; 47,XXY/46,XY; Hypergonadotropic profile; Micropenis; Testicular hypotrophy	Not reported in ClinVar	
rs751670386	0c.257A > T	p.Asp86Val	Spermatogenic failure; 47,XXY/46,XY; Hypergonadotropic profile; Testicular hypotrophy; Micropenis	Not reported in ClinVar	
rs1832458349	0c.247G > A	p.Val83Met	47,XXY/46,XY; Testicular hypotrophy; Micropenis	46,XY disorder of sex development	^29^

### 3.5 Genotype–phenotype correlation

Genotype–phenotype correlations analysis revealed a significant association between *NR5A1* variants and spermatogenic failure, the most frequent clinical feature, present in 60.8 % of patients.

A patient-level overview of *NR5A1* variants and their corresponding clinical manifestations is provided in Table 5, which highlights genotype–phenotype correlations and distinguishes novel from previously reported variants.

**Table 5:** Patient-level *NR5A1* variants and genotype–phenotype correlations.

Patient ID	*NR5A1* Variants (HGVS cDNA /Protein)	ACMG Classification	Predicted Pathogenicity	Observed Phenotype	Clinical Severity
1	0c.571C > T/p.Arg191Cys	P	Pathogenic	Spermatogenic failure with testicular hypotrophy	Severe
4	0c.532G > A/p.Gly178Arg	LP	Likely pathogenic	DSD features with spermatogenic failure (mosaic karyotype)	Very severe
7	0c.409G > T/p.Asp137Tyr	VUS	Possibly deleterious	Spermatogenic failure with testicular hypotrophy	Severe
7	0c.336G > T/p.Gln112His	VUS			
8	0c.584C > T/p.Ser195Phe	VUS	Potentially deleterious	Spermatogenic failure with testicular hypotrophy	Severe
9	0c.634G > A/p.Gly212Ser	P	Pathogenic/ Possibly deleterious	Spermatogenic failure	Severe
9	0c.336G > T/p.Gln112His	VUS			
9	0c.257A > T/p.Asp86Val	VUS			
18	0c.336G > T/p.Gln112His	VUS	Possibly deleterious	Spermatogenic failure (mild)	Moderate
19	0c.336G > T/p.Gln112His	VUS	Possibly deleterious	Spermatogenic failure with hypergonadotropic profile	Severe
19	0c.257A > T/p.Asp86Val	VUS			
20	0c.584C > T/p.Ser195Phe	VUS	Pathogenic/Possibly deleterious	Spermatogenic failure with elevated FSH/LH	Very severe
20	0c.571C > T/p.Arg191Cys	P			
20	0c.336G > T/p.Gln112His	VUS			
21	0c.386C > T/p.Pro129Leu	P	Pathogenic	DSD features with spermatogenic failure	Very severe
21	0c.368G > C/p.Gly123Ala	P			
22	0c.584C > T/p.Ser195Phe	VUS	Pathogenic/Likely pathogenic/Possibly deleterious	DSD features with spermatogenic failure	Very severe
22	0c.565C > T/p.Pro189Thr	LP			
22	0c.532G > A/p.Gly178Arg	LP			
22	0c.386C > T/p.Pro129Leu	P			
22	0c.368G > C/p.Gly123Ala	P			
22	0c.336G > T/p.Gln112His	VUS			
22	0c.257A > T/p.Asp86Val	VUS			
22	0c.247G > A/p.Val83Met	VUS			
23	0c.257A > T/p.Asp86Val	VUS	Possibly deleterious	DSD features with spermatogenic failure	Very severe

Variants are annotated according to HGVS nomenclature. ACMG classification follows the 2015 ACMG/AMP guidelines. ClinVar status is indicated as “Pathogenic”, “Likely pathogenic”, “VUS”, “Not reported”, or “Not reported (novel variant)”. P = Pathogenic; LP = Likely Pathogenic; VUS = Variant of Uncertain Significance.

**Fig. 3:**
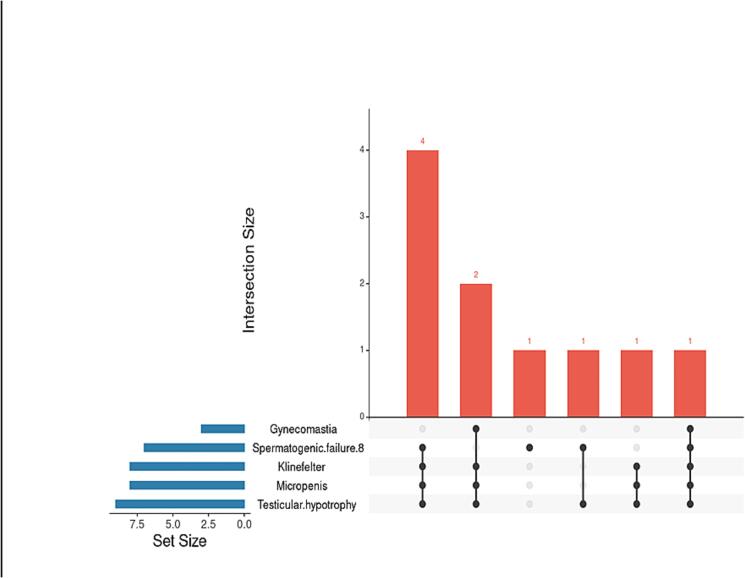
UpSet plot illustrating the co-occurrence of clinical phenotypes associated with genetic variants in the study cohort. The plot visualizes combinations of phenotypic traits observed among patients carrying deleterious *NR5A1* variants, highlighting frequent overlaps such as micropenis, testicular hypotrophy, and spermatogenic failure.

Phenotypic overlap was further illustrated with an UpSet plot (Fig. 3), showing micropenis, testicular hypotrophy, and spermatogenic failure as the predominant cluster of co-occurring features.

Patients carrying multiple *NR5A1* variants consistently exhibited more severe reproductive phenotypes (Table 5). For example, one patient with both the novel c.584C > T (p.Ser195Phe) and the pathogenic p.Arg191Cys variant presented with micropenis and azoospermia, while another patient carrying additional heterozygous variants alongside the novel variant presented severe DSD features, including micropenis, testicular hypotrophy, and azoospermia.

## 4 Discussion

Our study reinforces the critical role of *NR5A1* in male reproductive disorders, particularly in underrepresented populations. In this pilot senegalese cohort, *NR5A1* variants were detected in 83 % of patients. This unusually high frequency, compared to international reports, should be interpreted cautiously in light of the small sample size and exploratory design. But this high frequency highlighted a substantial genetic contribution to severe male infertility. Several of these variants have been previously reported in other ethnic groups, and their presence in West African individuals reinforces the global relevance of *NR5A1* in reproductive health and highlights the importance of including genetically diverse populations in genomic research.

We identified a novel heterozygous missense variant, c.584C > T (p.Ser195Phe), located in the hinge region of SF-1, a domain essential for nuclear localization, protein flexibility and protein–protein interactions.^30^, ^31^ The affected residue, serine at position 195, is highly conserved across species, and the variant is absent from major public databases (gnomAD, dbSNP, ClinVar). In silico predictions classified the substitution as deleterious, suggesting potential disruption of protein folding or interactions. Clinically, p.Ser195Phe was associated with micropenis, testicular hypotrophy, and azoospermia, consistent with gonadal dysgenesis. Among the three patients carrying this variant, Patient 8 harbored it alone, Patient 20 also carried p.Arg191Cys, and Patient 22 (with DSD) carried multiple *NR5A1* variants, including p.Pro129Leu, p.Gly123Ala, and five additional pathogenic variants. These observations suggested that the co-occurrence of several variants in the same individual may have a cumulative or oligogenic effect, as previously proposed in the literature. However, functional validation will be required to confirm this hypothesis. These observations were in line with previous reports implicating hinge-region variants in 46,XY DSD and impaired spermatogenesis.^23^, ^30^

After applying variant filtering, a total of eleven *NR5A1* variants were retained in our cohort, including the novel p.Ser195Phe. Seven of these variants have been previously described in the literature, while three were identified only in public databases and predicted as potentially pathogenic. This comprehensive profile highlights the diversity of *NR5A1* variation in Senegalese men with severe reproductive disorders.

Our findings supported cumulative or oligogenic effects in *NR5A1*-related pathogenesis. Patients carrying multiple variants consistently exhibited more severe phenotypes, particularly the triad of micropenis, testicular hypotrophy, and azoospermia, illustrating additive or synergistic disruption of SF-1 function. Notably, co-occurring variants such as p.Gly123Ala/p.Pro129Leu and p.Ser195Phe/p.Arg191Cys were associated with the most pronounced phenotypes, consistent with the oligogenic model proposed by Kouri et al.^32^ These observations underscore the importance of evaluating combined genotypes when assessing clinical severity in underrepresented populations.

Comparing our results with the literature, the seven previously reported variants in our cohort showed consistent phenotypic associations. p.Gly212Ser (rs201095702) and p.Arg191Cys (rs1253324106) have been linked to spermatogenic failure in multiple studies,^23^, ^24^ while p.Pro189Thr (rs1483691434) and p.Gly178Arg (rs543895681) have been reported in cases of gonadal dysgenesis, micropenis, and variable sex development.^25^, ^26^ The variants p.Pro129Leu (rs200749741) and p.Gly123Ala (rs200163795) have also been associated with premature ovarian failure and spermatogenic defects in African ancestry cohorts,^23^, ^27^, ^28^ aligning with the severe phenotypes observed in our patients. Finally, p.Val83Met (rs1832458349) was previously reported in 46,XY disorders of sex development,^29^ supporting its pathogenic potential. Collectively, these comparisons demonstrate that our findings are consistent with international reports and emphasize the global relevance of *NR5A1* in reproductive health.

Phenotypically, micropenis was observed in carriers of p.Ser195Phe, p.Gly212Ser, and p.Arg191Cys; testicular hypotrophy in p.Ser195Phe and p.Gly178Arg; and azoospermia in p.Ser195Phe, p.Pro129Leu, and p.Gly123Ala. The heterogeneity of clinical features, including co-occurring DSD and spermatogenic failure, highlights the variable expressivity and pleiotropy of *NR5A1* variants, mirroring patterns reported in prior studies.^19^, ^20^, ^26^, ^33^ While parallels with the Testicular Dysgenesis Syndrome model can be drawn, it remains premature to establish a direct causal link.^33^ The diversity and co-occurrence of clinical features, illustrated by the UpSet plot, underscore the need for variant-specific clinical evaluation and functional validation. Moreover, the absence of *NR5A1* variants in four patients despite similar phenotypes emphasizes the genetic heterogeneity of male infertility and the potential contribution of additional genes or environmental factors, reinforcing the value of broader genetic studies in underrepresented populations.

## 5 Limitations

This study has several limitations inherent to its pilot design. The small sample size of patients (n = 23) and the very small control group (n = 2) limits statistical power and generalizability of the findings. Larger multicenter cohorts will be required to validate genotype–phenotype correlations and provide more accurate estimates of *NR5A1* variant prevalence.Additionally, no functional validation experiments were performed, which is particularly important for the novel variant p.Ser195Phe. Segregation analyses and hormonal profiling of unaffected family members were not conducted, which would have strengthened interpretation of inheritance patterns.Despite these limitations, this study provides valuable preliminary insights into *NR5A1* variation in a West African population and underscores the importance of including African cohorts in reproductive genetics research.

## 6 Conclusions

This study reveals notable genetic diversity in the *NR5A1* gene among infertile Senegalese men, reinforcing its critical role in human spermatogenesis and sexual development. A novel heterozygous missense variant, c.584C > T (p.Ser195Phe), located in a highly conserved region of the SF-1 protein, was identified and found to be associated with severe infertility phenotypes, including micropenis, testicular hypotrophy, and azoospermia.

These findings emphasized the importance of extending genomic research to underrepresented populations, such as those in sub-Saharan Africa. They also provided valuable new insights into the genetic underpinnings of male infertility. Integrating *NR5A1* into targeted genetic screening may improve diagnostic accuracy and inform personalized management strategies in affected individuals.

## Ethical approval

This study was approved by the Research Ethics Committee of Cheikh Anta Diop University, Dakar, Senegal (approval number CER/UCAD/AD/MsN/014/2020; issued on February 17, 2021). All procedures were conducted in accordance with the Declaration of Helsinki and adhered to national and institutional ethical guidelines. Written informed consent was obtained from all participants following a detailed explanation of the study objectives, procedures, and potential risks.

## CRediT authorship contribution statement

**Adji Dieynaba Diallo:** Writing – review & editing, Writing – original draft, Methodology, Investigation, Funding acquisition, Formal analysis, Data curation, Conceptualization. **Arame Ndiaye:** Writing – review & editing, Validation, Supervision. **Ndiaga Diop:** Writing – review & editing. **Fatou Diop Gueye:** Writing – review & editing, Software. **Mame Venus Gueye:** Writing – review & editing. **Yacouba Dia:** Writing – review & editing, Software. **Amath Thiam:** Writing – review & editing, Resources. **Abdoulaye Séga Diallo:** Writing – review & editing, Resources. **Rokhaya Ndiaye:** Writing – review & editing. **Oumar Faye:** Writing – review & editing, Supervision. **Mama Sy:** Writing – review & editing, Validation, Supervision.

## Declaration of competing interest

The authors declare that they have no known competing financial interests or personal relationships that could have appeared to influence the work reported in this paper.

## References

[1] World Health Organization. Infertility Prevalence Estimates, 1990–2021; WHO: Geneva, Switzerland; 2023. [Licence: CC BY-NC-SA 3.0 IGO].

[2] Faye O., Moreau J.C., Agonhessou S.M. (2000). Intérêt des tests post-coïtaux et cyto-spermiologiques dans l’exploration de la stérilité conjugale au Sénégal. Dakar Med.

[3] Levine H., Jørgensen N., Martino-Andrade A. (2023). Temporal trends in sperm count: a systematic review and meta-regression analysis of samples collected globally in the 20th and 21st centuries. Hum Reprod Update.

[4] Rolland M., Le Moal J., Wagner V. (2013). Decline in semen concentration and morphology in a sample of 26,609 men close to general population between 1989 and 2005 in France. Hum Reprod.

[5] Singh K., Jaiswal D. (2011). Human male infertility. Reprod Sci.

[6] Luppino G., Wasniewska M., Coco R. (2024). Role of NR5A1 gene mutations in disorders of sex development: molecular and clinical features. Curr Issues Mol Biol.

[7] Morais da Silva S., Hacker A., Harley V. (1996). Sox9 expression during gonadal development implies a conserved role in testis differentiation. Nat Genet.

[8] Sekido R., Lovell-Badge R. (2008). Sex determination involves synergistic action of SRY and SF1 on a specific Sox9 enhancer. Nature.

[9] Shen W.H., Moore C.C., Ikeda Y., Parker K.L., Ingraham H.A. (1994). SF-1 regulates the Müllerian inhibiting substance gene. Cell.

[10] Watanabe K., Clarke T.R., Lane A.H. (2000). Endogenous expression of MIS in postnatal Sertoli cells. PNAS.

[11] Leers-Sucheta S., Morohashi K., Mason J.I., Melner M.H. (1997). SF-1 activation of 3β-HSD promoter. J Biol Chem.

[12] Sugawara T., Kiriakidou M., McAllister J.M. (1997). SF-1 binding in the STAR promoter. Biochemistry.

[13] Takayama K., Sasano H., Fukaya T. (1995). Immunolocalization of Ad4BP in human ovaries. J Clin Endocrinol Metab.

[14] Domenice S., Machado A.Z., Ferreira F.M. (2016). Wide spectrum of NR5A1 phenotypes in 46,XY and 46,XX. Birth Defects Res C Embryo Today.

[15] Fabbri-Scallet H., de Sousa L.M., Maciel-Guerra A.T. (2020). Mutation update for NR5A1 in DSD and infertility. Hum Mutat.

[16] Camats N., Pandey A.V., Fernández-Cancio M. (2012). Ten novel NR5A1 mutations in 46,XY DSD. J Clin Endocrinol Metab.

[17] Werner R., Mönig I., Lünstedt R. (2017). New NR5A1 mutations in gonadal dysgenesis. PLoS One.

[18] Camats N., Fernández-Cancio M., Audí L. (2018). Oligogenic origin of NR5A1-related DSD. Eur J Hum Genet.

[19] Elzenaty R.N., Martinez de Lapiscina I., Kouri C. (2025). SF1next study: 35 novel NR5A1 variants. J Clin Endocrinol Metab.

[20] Kouri C., Sommer G., Martinez de Lapiscina I. (2024). NR5A1 variant cohort study. EBioMedicine.

[21] World Health Organization. WHO laboratory manual for the examination and processing of human semen (5^e^ édition). Genève, Suisse : World Health Organization; 2010.

[22] Sudhakar D.V.S., Nizamuddin S., Manisha G. (2017). NR5A1 mutations are not associated with male infertility in Indian men. Andrologia.

[23] Bashamboo A., Ferraz-de-Souza B., Lourenço D. (2010). Human male infertility associated with mutations in NR5A1 encoding steroidogenic factor 1. Am J Hum Genet.

[24] Wang A., Zhang H., Wang L.N. (2018). Next-generation sequencing reveals genetic landscape in 46,XY disorders of sexual development patients with variable phenotypes. Hum Genet.

[25] Luo W., Ke H., Tang S. (2023). Next-generation sequencing of 500 POI patients identified novel responsible monogenic and oligogenic variants. J. Ovarian Res..

[26] Eggers S., Sadedin S., van den Bergen J.A. (2016). Disorders of sex development: insights from targeted gene sequencing of a large international patient cohort. Genome Biol.

[27] Lourenço D., Brauner R., Lin L. (2009). Mutations in NR5A1 associated with ovarian insufficiency. N Engl J Med.

[28] Voican A., Bachelot A., Bouligand J. (2013). NR5A1 (SF-1) mutations are not a major cause of primary ovarian insufficiency. J Clin Endocrinol Metab.

[29] Song Y., Fan L., Gong C. (2018). Phenotype and molecular characterizations of 30 children from China with NR5A1 mutations. Front Pharmacol.

[30] Ferraz-de-Souza B. (2011). Steroidogenic factor-1 (SF-1, NR5A1) mutations: an expanding spectrum of phenotypes. Horm Res Paediatr.

[31] Robevska G. (2018). Functional characterization of novel NR5A1 variants reveals multiple complex roles in disorders of sex development. Hum Mutat.

[32] Kouri C., Naamneh-Elzenaty R., de Lapiscina I.M., Flück C.E. (2025). Broader impact and outcome of human NR5A1/SF1 variants. Best Pract Res Clin Endocrinol Metab.

[33] Skakkebæk N.E., Lindahl-Jacobsen R., Levine H. (2022). Environmental factors in declining human fertility. Nat Rev Endocrinol.

