# Supplementary material for: NR5A1 gene variants in infertile Senegalese men: Discovery of a novel missense variant and genotype-phenotype correlation
Source: J Genet Eng Biotechnol. 2025 Sep 27;23(4):100578. doi: 10.1016/j.jgeb.2025.100578 (PMC12510190; doi:10.1016/j.jgeb.2025.100578)
Supplement: Supplementary Data 1 [file mmc1.docx]

Supplementary Table S1. Sequence of primers used to amplify coding region (exons 2 to 7) of NR5A1

| **Exons** | **Forward primers** | **Reverse primers** | **Tm (°C)** | **Amplicon**  **size (bp)** |
| --- | --- | --- | --- | --- |
| 2 and 3 | AACGAATCCCAATCGAAGCG | TTGGATACTTCTCAGCCCAACC | 57 | 861 |
| 4 | GTGTTGAGCAGGGGAGAGAG | AGAGAAGGGCTCTGGGTAGC | 60 | 520 |
| 5 | ATCTGGGTAGATGGGCACAG | GTGGGGAAAGGGCTGATAAT | 60 | 395 |
| 6 | TCTGACCTGCACCTCCAATC | CTCTGGCTGTCTCCACCTCT | 58 | 301 |
| 7 | ATGCCCATGTCTTTGATGGT | GGTGGGCATCAGAAAATGAA | 58 | 475 |
