# Supplementary material for: NR5A1 gene variants in infertile Senegalese men: Discovery of a novel missense variant and genotype-phenotype correlation
Source: J Genet Eng Biotechnol. 2025 Sep 27;23(4):100578. doi: 10.1016/j.jgeb.2025.100578 (PMC12510190; doi:10.1016/j.jgeb.2025.100578)
Supplement: Supplementary Data 2 [file mmc2.docx]

Supplementary Table S2. Clinical, demographic, hormonal, and karyotype characteristics of infertile Senegalese men and fertile controls.

| **ID** | **Sex/ Gender** | **Age (years)** | **BMI (kg/m²)** | **Consanguinity/ Ethnicity** | **Clinical Signs** | **Sperm Analysis** | **Hormonal Profile** | **Karyotype** | **Remarks** |
| --- | --- | --- | --- | --- | --- | --- | --- | --- | --- |
| C1 | M/M | 28 | 22.5 | No/Ouolof | Normal | Normozoospermia | FSH: 5.0; LH: 4.5; T: 18 | 46,XY | Control |
| C2 | M/M | 32 | 23 | No/Sérère | Normal | Normozoospermia | FSH: 5.5; LH: 4.0; T: 17 | 46,XY | Control |
| 1 | M/M | 32 | 25.5 | No/Halpulaar | Testicular hypotrophy | Azoospermia | FSH: 33.80; LH: 17.71; T: 3.24 | 46,XY |  |
| 2 | M/M | 29 | 28.7 | Yes/Ouolof | Gynecomastia; Testicular hypotrophy | Azoospermia | FSH: 64; LH: 68; T: 19.8 | 46,XY |  |
| 3 | M/M | 40 | 20.1 | No/Ouolof | Testicular hypotrophy | Azoospermia | FSH: 14.71; T: 12.75 | 46,XY | Family history of infertility (brother) |
| 4 | M/DSD | 34 | 25.3 | No/Halpulaar | Gynecomastia; Hypospadias; Testicular hypotrophy; Micropenis | Azoospermia | FSH: 58.28; T: 14.78 | 47, XXY/46, XX | Confirmed DSD |
| 5 | M/M | 30 | 20.5 | No/Ouolof | Normal | Azoospermia | Not reported | 46,XY |  |
| 6 | M/M | 30 | 24.7 | Yes/Halpulaar | Normal | Cryptozoospermia | Not reported | 46,XY |  |
| 7 | M/M | 30 | 23.8 | Yes/Diola | Testicular hypotrophy | Azoospermia | FSH: 27.60; LH: 19.15; T: 15.26 | 46,XY |  |
| 8 | M/M | 30 | 25 | No/Ouolof | Testicular hypotrophy | Azoospermia | FSH: 23.68; T: 5.73 | 46,XY |  |
| 9 | M/M | 44 | 21.4 | No/Sérère | Normal | Severe oligozoospermia | FSH: 39.70; T: 6.82 | 46,XY |  |
| 10 | M/M | 32 | 21.3 | No/Sérère | Normal | Cryptozoospermia | Not reported | 46,XY |  |
| 11 | M/M | 22 | 16.6 | No/Sarakhole | Normal | Azoospermia | Not reported | 46,XY |  |
| 12 | M/M | 31 | 17.4 | Yes/Halpulaar | Testicular hypotrophy | Cryptozoospermia | FSH: 32.8; T: 8.8 | 46,XY |  |
| 13 | M/M | 39 | 16.1 | Yes/Halpulaar | Normal | Azoospermia | Not reported | 46,XY |  |
| 14 | M/M | 45 | 24.4 | No/Diola | Normal | Severe oligozoospermia | Not reported | 46,XY |  |
| 15 | M/M | 23 | 20.8 | No/Bambara | Normal | Azoospermia | Not reported | 46,XY |  |
| 16 | M/M | 23 | 20.5 | Yes/Halpulaar | Normal | Severe oligozoospermia | FSH: 16.2; T: 8.8 | 46,XY |  |
| 17 | M/M | 25 | 23 | Yes/Ouolof | Normal | Azoospermia | FSH: 41.73; LH: 11.4; T: 1.34 | 46,XY |  |
| 18 | M/M | 29 | 22.2 | No/Diola | Normal | Cryptozoospermia | Not reported | 46,XY |  |
| 19 | M/M | 30 | 23.8 | No/Sérère | Normal | Azoospermia | FSH: 39.70; T: 1.42 | 46,XY |  |
| 20 | M/M | 28 | 22.3 | No/Sérère | Normal | Azoospermia | FSH: 29.56; LH: 17.44; T: 2.04 | 46,XY |  |
| 21 | M/DSD | 21 | 21.5 | Yes/Ouolof | Testicular hypotrophy; Gynecomastia | Azoospermia | FSH: 24.34; LH: 18.42; T: 0.35 | 47,XXY/46,XY | Confirmed DSD |
| 22 | M/DSD | 38 | 26.7 | Yes/Sérère | Testicular hypotrophy; Micropenis | Azoospermia | FSH: 10.71; T: 16.51 | 47,XXY/46,XY | Confirmed DSD |
| 23 | M/DSD | 30 | 18.5 | Yes/Ouolof | Testicular hypotrophy; Inguinal hernia | Azoospermia | FSH: 23.62; LH: 24.45; T: 0.68 | 47,XXY/46,XY | Confirmed DSD |
| Mean± SD (Patients, n=23) |  | 31.1± 6.5 |  |  |  |  |  |  |  |
| Mean± SD (Controls, n=2) |  | 30.0± 2.8 |  |  |  |  |  |  |  |

FSH = follicle-stimulating hormone; LH = luteinizing hormone; T = testosterone; BMI = body mass index; DSD = disorder of sex development
