# Supplementary material for: NR5A1 gene variants in infertile Senegalese men: Discovery of a novel missense variant and genotype-phenotype correlation
Source: J Genet Eng Biotechnol. 2025 Sep 27;23(4):100578. doi: 10.1016/j.jgeb.2025.100578 (PMC12510190; doi:10.1016/j.jgeb.2025.100578)
Supplement: Supplementary Data 3 [file mmc3.docx]

Supplementary Table S3. In silico predictions of all NR5A1 variants identified in the Senegalese cohort

| **HGVSc** | **HGVSp** | **Variant effect** | **Rs number** | **ACMG Class.** | **Clinvar** | **SIFT** | **PolyPhen-2** | **MutationTaste** | **REVEL** | **E-SNPs&GO** | **M-CAP** | **Panther** | **SNPs&Go** |
| --- | --- | --- | --- | --- | --- | --- | --- | --- | --- | --- | --- | --- | --- |
| c.645G>A | p.Glu215= | Synonymous | rs1832447270 | **VUS** | ND | ND | B | B | LB | ND | ND | ND | ND |
| c.634G>A | p.Gly212Ser | Missense | rs201095702 | **P** | **P** | T | B | P | likely disease causing | B | P | Posdam | Neu |
| c.626T>A | p.Leu209Gln | Missense | rs1205624250 | **VUS** | ND | ND | B | B | LB | B | P | Prben | Neu |
| c.613C>G | p.Pro205Ala | Missense | rs755459988 | **VUS** | ND | ND | B | B | LB | B | P | Prdam | Neu |
| c.608G>T | p.Ser203Ile | Missense | rs770165012 | **VUS** | ND | ND | B | B | LB | B | P | Prdam | Neu |
| c.603T>C | p.Tyr201= | Synonymous | rs1389543537 | **B** | ND | ND | ND | B | ND | ND | ND | ND | ND |
| c.594G>A | p.Pro198= | Synonymous | rs142414614 | **B** | **B** | T | ND | B | ND | ND | ND | ND | ND |
| c.584C>T | p.Ser195Phe | Missense | novel variant | **VUS** | ND |  | Prdam | P | ND | P | P | Prdam | Neu |
| c.571C>T | p.Arg191Cys | Missense | rs1253324106 | **P** | ND | ND | Posdam | P | likely disease causing | P | P | Prdam | Dis |
| c.565C>T | p.Pro189Thr | Missense | rs1483691434 | **LP** | **LP** | ND | B | B | LB | B | P | Posdam | Neu |
| c.552C>T | p.Leu184= | Synonymous | rs1199145590 | **VUS** | ND | ND | ND | B | ND | ND | ND | ND | ND |
| c.532G>A | p.Gly178Arg | Missense | rs543895681 | **LP** | ND | ND | Prdam | B | LB | B | P | Posdam | Dis |
| c.516C>T | p.Ala172= | Synonymous | rs113506523 | **VUS** | **B** | T | ND | B | LB | ND | ND | ND | ND |
| c.487G>A | p.Asp163Asn | Missense | rs377294547 | **VUS** | ND | T | B | B | LB | B | P | Posdam | Dis |
| c.437G>C | p.Gly146Ala | Missense | rs1110061 | **B** | **B** | T | B | B | LB | B | P | Prdam | Neu |
| c.409G>T | p.Asp137Tyr | Missense | rs745949372 | **VUS** | ND | ND | Posdam | B | LB | P | P | Prdam | Neu |
| c.386C>T | p.Pro129Leu | Missense | rs200749741 | **P** | **P** | T | B | B | LB | B | P | Posdam | Neu |
| c.375G>A | p.Pro125= | Synonymous | rs1110062 | **B** | **B** | T | ND | B | LB | ND | ND | ND | ND |
| c.368G>C | p.Gly123Ala | Missense | rs200163795 | **P** | **P** | T | B | B | LB | B | P | Posdam | Neu |
| c.363G>A | p.Glu121= | Synonymous | rs770608642 | **VUS** | ND | ND | ND | B | LB | ND | ND | ND | ND |
| c.339T>A | p.Ile113= | Synonymous | rs1588622157 | **LB** | **LB** | ND | ND | B | ND | ND | ND | ND | ND |
| c.336G>T | p.Gln112His | Missense | rs1564152710 | **VUS** | ND | ND | B | B | LB | P | P | Prben | Neu |
| c.257A>T | p.Asp86Val | Missense | rs751670386 | **VUS** | ND | ND | Prdam | P | LB | P | P | Prdam | Dis |
| c.247G>A | p.Val83Met | Missense | rs1832458349 | **VUS** | ND | ND | Prdam | P | ND | P | P | Prdam | Dis |
| c.871-20C>T |  | Intron Variant | rs2297605 | **B** | **B** | ND | ND | B | ND | ND | ND | ND | ND |
| c.610-13C>T |  | Intron Variant | rs189724865 | **B** | **B** | ND | ND | B | ND | ND | ND | ND | ND |

ACMG, American College of Medical Genetics and Genomics Classification ; B, Benign; Dis, disease; Het, Heterozygous; Hom, Homozygous; LB, Likely Benign; LPLikely Pathogenic; MAF, Minor allele frequency corresponding to the highest MAF within the different populations in gnoMAD; ND, Not Defined; Neu, Neutral; P, Pathogenic; gnoMAD, genome aggregation database; Prben, probably benign; Prdam, probably damaging, Psdam, possibly damaging; rs, Reference, VUS, Variant of unknown significance; In silico analysis with webtool: SIFT (https://sift.bii.a-star.edu.sg/), PolyPhen-2 (http://genetics.bwh.harvard.edu/pph2/), MutationTaster (https://www.genecascade.org/MutationTaster2021/#transcript ), Panther (http://pantherdb.org/ ), SNPs&Go (https://snps-and-go.biocomp.unibo.it/snps-and-go/ ), M-CAP (http://bejerano.stanford.edu/mcap/) , REVEL (https://sites.google.com/site/revelgenomics/ ), E-SNPs&GO (https://esnpsandgo.biocomp.unibo.it/ ) *NR5A1*: NM_004959.5
