# Supplementary material for: NR5A1 gene variants in infertile Senegalese men: Discovery of a novel missense variant and genotype-phenotype correlation
Source: J Genet Eng Biotechnol. 2025 Sep 27;23(4):100578. doi: 10.1016/j.jgeb.2025.100578 (PMC12510190; doi:10.1016/j.jgeb.2025.100578)
Supplement: Supplementary Data 4 [file mmc4.docx]

Supplementary Table S4. Allelic variants of NR5A1/SF-1 and population frequencies in a Senegalese male infertility cohort

| **Variant ID** | **Allele** | **Exon** | **Domaine** | **HGVS Consequence** | | **Rs number** | **MAF (cohort)**  **P < 0,001** | **MAF (gnomAD v2.1.1/ dbSNP)** | | |
| --- | --- | --- | --- | --- | --- | --- | --- | --- | --- | --- |
|  |  |  |  | **HGVSc** | **HGVSp** |  |  | **AFR** | **EUR** | **EAS** |
| 9 :127262594 C>T | C>T | 4 | Hinge | c.645G>A | p.Glu215= | rs1832447270 | 0,130 | NA^1^ | NA | NA |
| 9 :127262605 C>T | C>T | 4 | Hinge | c.634G>A | p.Gly212Ser | rs201095702 | 0,022 | 0,000 | 0.00002628 | 0.001726 |
| 9 :127262613 A>T | A>T | 4 | Hinge | c.626T>A | p.Leu209Gln | rs1205624250 | 0,043 | 0.000 | 0.000 | 0.000 |
| 9 :127262626 G>C | G>C | 4 | Hinge | c.613C>G | p.Pro205Ala | rs755459988 | 0,043 | 0,000 | 0,000 | 0,000 |
| 9 :127262631 C>A | C>A | 4 | Hinge | c.608G>T | p.Ser203Ile | rs770165012 | 0,022 | 0,000 | 0,00001689 | 0,000 |
| 9 :127262636 A>G | A>G | 4 | Hinge | c.603T>C | p.Tyr201= | rs1389543537 | 0,022 | 0,000 | 0,00001682 | 0,000 |
| 9 :127262645 C>T | C>T | 4 | Hinge | c.594G>A | p.Pro198= | rs142414614 | 0,043 | 0,04125 | 0,00006698 | 0,000 |
| **9 :127262655 G>A** | **G>A** | **4** | Hinge | **c.584C>T** | **p.Ser195Phe** | **-** | **0,065** | **NA** | **NA** | **NA** |
| 9 :127262668 G>A | G>A | 4 | Hinge | c.571C>T | p.Arg191Cys | rs1253324106 | 0,043 | 0,000 | 0,00002618 | 0,000 |
| 9 :127262674 G>T | G>T | 4 | Hinge | c.565C>A | p.Pro189Thr | rs1483691434 | 0,022 | 0,000 | 0,000 | 0,000 |
| 9 :127262687 G>A | G>A | 4 | Hinge | c.552C>T | p.Leu184= | rs1199145590 | 0,022 | 0,000 | 0,000 | 0,000 |
| 9 :127262707 C>T | C>T | 4 | Hinge | c.532G>A | p.Gly178Arg | rs543895681 | 0,043 | 0,00005491 | 0,00001157 | 0,000 |
| 9 :127262723 G>A | G>A | 4 | Hinge | c.516C>T | p.Ala172= | rs113506523 | 0,174 | 0,03370 | 0,00004162 | 0,000 |
| 9 :127262752 C>T | C>T | 4 | Hinge | c.487G>A | p.Asp163Asn | rs377294547 | 0,022 | 0,000 | 0,00007476 | 0,00006172 |
| 9 :127262802 C>G | C>G | 4 | Hinge | c.437G>C | p.Gly146Ala | rs1110061 | 0,826 | 0,7568 | 0,01187 | 0,3465 |
| 9 :127262830 C>A | C>A | 4 | Hinge | c.409G>T | p.Asp137Tyr | rs745949372 | 0,022 | 0,000 | 0,000 | 0,000 |
| 9 :127262853 G>A | G>A | 4 | Hinge | c.386C>T | p.Pro129Leu | rs200749741 | 0,087 | 0,002474 | 0,00004087 | 0,000 |
| 9 :127262864 C>T | C>T | 4 | Hinge | c.375G>A | p.Pro125= | rs1110062 | 0,522 | 0,000 | 0,00003276 | 0,00003290 |
| 9 :127262871C>G | C>G | 4 | Hinge | c.368G>C | p.Gly123Ala | rs200163795 | 0,087 | 0,002496 | 0,00004069 | 0,000 |
| 9 :127262876 C>T | C>T | 4 | Hinge | c.363G>A | p.Glu121= | rs770608642 | 0,043 | 0,000 | 0,000009266 | 0,00005588 |
| 9: 127262900 A>T | A>T | 4 | Hinge | c.339T>A | p.Ile113= | rs1588622157 | 0,022 | NA | NA | NA |
| 9 :127262903 C>A | C>A | 4 | Hinge | c.336G>T | p.Gln112His | rs1564152710 | 0,130 | 0,000 | 0,000009180 | 0,000 |
| 9 :127262982 T>A | T>A | 4 | DBD | c.257A>T | p.Asp86Val | rs751670386 | 0,109 | 0,000 | 0,000 | 0,000 |
| 9 :127262992 C>T | C>T | 4 | DBD | c.247G>A | p.Val83Met | rs1832458349 | 0,022 | 0,000 | 0,000 | 0,000 |
| 9 :127255448 G>A | G>A | 5 | LBD | c.871-20C>T |  | rs2297605 | 0,174 | 0,000 | 0,00002752 | 0,000 |
| 9 :127255441 G>A | G>A | 5 | LBD | c.871-13C>T |  | rs189724865 | 0,043 | 0,01255 | 0,00005589 | 0,00005049 |

^1^ NA = data not available in the database.
