# Supplementary material for: NR5A1 gene variants in infertile Senegalese men: Discovery of a novel missense variant and genotype-phenotype correlation
Source: J Genet Eng Biotechnol. 2025 Sep 27;23(4):100578. doi: 10.1016/j.jgeb.2025.100578 (PMC12510190; doi:10.1016/j.jgeb.2025.100578)
Supplement: Supplementary Data 5 [file mmc5.docx]

Supplementary Table S5. Clinical, hormonal, and genetic characteristics of infertile patients without detectable NR5A1 variants.

| **ID** | **Age (years)** | **Ethnicity** | **Clinical Signs** | **Semen category** | **Hormonal Profile** | | | **Karyotype** | **Phenotype** | **Severity grade** |
| --- | --- | --- | --- | --- | --- | --- | --- | --- | --- | --- |
|  |  |  |  |  | FSH (IU/L) | LH (IU/L) | Total T (ng/mL) |  |  |  |
| 10 | 32 | Sérère | Normal genital exam | Cryptozoospermia | NR | NR | NR | 46,XY | Spermatogenic failure (mild) | Moderate |
| 11 | 22 | Sarakhole | Normal genital exam | Azoospermia | NR | NR | NR | 46,XY | Spermatogenic failure | Severe |
| 12 | 31 | Halpulaar | Testicular hypotrophy | Cryptozoospermia | 32.8 | NR | 8.8 | 46,XY | Spermatogenic failure (mild) | Moderate |
| 15 | 23 | Bambara | Normal genital exam | Azoospermia | NR | NR | NR | 46,XY | Spermatogenic failure | Severe |
